# An Insight into the Structural Requirements and Pharmacophore Identification of Carbonic Anhydrase Inhibitors to Combat Oxidative Stress at High Altitudes: An In-Silico Approach

**DOI:** 10.3390/cimb44030068

**Published:** 2022-02-23

**Authors:** Amena Ali, Abuzer Ali, Musarrat Husain Warsi, Mohammad Akhlaquer Rahman, Mohamed Jawed Ahsan, Faizul Azam

**Affiliations:** 1 High Altitude Research Center, Taif University, P.O. Box 11099, Taif 21944, Saudi Arabia; 2 Department of Pharmaceutical Chemistry, College of Pharmacy, Taif University, P.O. Box 11099, Taif 21944, Saudi Arabia; 3 Department of Pharmacognosy, College of Pharmacy, Taif University, P.O. Box 11099, Taif 21944, Saudi Arabia; abuali@tu.edu.sa; 4 Department of Pharmaceutics and Industrial Pharmacy, College of Pharmacy, Taif University, P.O. Box 11099, Taif 21944, Saudi Arabia; mvarsi@tu.edu.sa (M.H.W.); mrahman@tu.edu.sa (M.A.R.); 5 Department of Pharmaceutical Chemistry, Maharishi Arvind College of Pharmacy, Ambabari Circle, Jaipur 302 039, Rajasthan, India; jawedpharma@gmail.com; 6 Department of Pharmaceutical Chemistry & Pharmacognosy, Unaizah College of Pharmacy, Qassim University, P.O. Box 5888, Unaizah 51911, Saudi Arabia; f.azam@qu.edu.sa

**Keywords:** 1,3,4-thiadiazole, oxidative stress, molecular modeling, carbonic anhydrase (CA) inhibitor, high altitude

## Abstract

Carbonic anhydrases (CA) inhibitory action could be linked to the treatment of a number of ailments, including cancer, osteoporosis, glaucoma, and several neurological problems. For the development of effective CA inhibitors, a variety of heterocyclic rings have been investigated. Furthermore, at high altitudes, oxygen pressure drops, resulting in the formation of reactive oxygen and nitrogen species, and CA inhibitors having role in combating this oxidative stress. Acetazolamide contains thiadiazole ring, which has aroused researchers’ interest because of its CA inhibitory action. In the present study, we used a number of drug design tools, such as pharmacophore modeling, 3D QSAR, docking, and virtual screening on twenty-seven 1,3,4-thiadiazole derivatives that have been described as potential CA inhibitors in the literature. An atom-based 3D-QSAR analysis was carried out to determine the contribution of individual atoms to model generation, while a pharmacophore mapping investigation was carried out to find the common unique pharmacophoric properties required for biological activity. The coefficient of determination for both the training and test sets were statistically significant in the generated model. The best QSAR model was chosen based on the values of R^2^ (0.8757) and Q^2^ (0.7888). A molecular docking study was also conducted against the most potent analogue **4m**, which has the highest SP docking score (−5.217) (PDB ID: 6g3v). The virtual screening revealed a number of promising compounds. The screened compound ZINC77699643 interacted with the amino acid residues, Pro201 and Thr199, in the virtual screening study (PDB ID: 6g3v). These interactions demonstrated the significance of the CA inhibitory activity of the compound. Furthermore, ADME study revealed useful information regarding compound’s drug-like properties. Therefore, the findings of the present investigation could aid in the development of more potent CA inhibitors, which could benefit the treatment of oxidative stress at high altitudes.

## 1. Introduction

At high altitudes, oxygen pressure drops, resulting in the formation of reactive oxygen and nitrogen species (RONS), which have been linked to a variety of oxidative stress related diseases [1,2]. The formation of reactive oxygen and nitrogen species (RONS) causes oxidative damage to biomolecules such as protein and DNA, which has been linked to a number of diseases [3,4,5,6]. At high altitude, the body’s antioxidant enzymes system becomes unresponsive. In addition, at this height, the RONS-producing sources, which include various mitochondrial transport systems, nitric oxide synthase, and xanthine oxidase, become activated [7,8,9,10]. Physical activity can sometimes cause an increase in oxidative stress, fatigue, headaches, nausea, anorexia, and poor sleep. A number of medications are available to treat oxidative stress, including carbonic anhydrase (CA) inhibitors [11] and, in particular, acetazolamide [12]. The literature survey revealed that acetazolamide (a specific inhibitor of CA) is used to treat high-altitude pulmonary oedema (HAPE) [13].

Carbonic anhydrase enzyme of various types is found in prokaryotes and eukaryotes. Carbonic anhydrases (CAs, EC 4.2.1.1) encode bacteria found in three different genetic families, the α-, β-, and γ-classes. These metalloenzymes interfere with pH regulation and other important physiological processes in these organisms by equilibrating CO2 and bicarbonate. These enzymes differ in terms of tissue expression, kinetic properties, and localization in different parts of the body. CAs I, II, III, VII, and XIII are cytosolic carbonic enzymes, whereas CAs IV, IX, XII, and XIV are cell membrane associated enzymes [14]. CAs VA and VB are found near the mitochondrial region, while VI is found in saliva and milk. Catalytic activity is absent in CAs VIII, X, and XI. CAs play various roles in our bodies, including electrolyte balance in various organs, biosynthetic reactions, calcification, bone resorption, pH and CO_2_ homeostasis, and tumorigenicity.

CAs’ inhibitory activity has been linked to the treatment of a number of diseases, including cancer, osteoporosis, glaucoma, and some neurological disorders [15]. The use of heterocyclic rings in the development of powerful CA inhibitors has been studied. The thiadiazole (Acetazolamide) ring has piqued scientists’ interest due to its ability to inhibit a variety of diseases [16,17].

In the current study, we used 3D QSAR to identify the structural requirements of 1,3,4-thiadiazole derivatives for potential human CA-I inhibitions. The presence of various pharmacophoric features such as aromatic groups, electron donating groups, electron withdrawing groups, and hydrophobic long chains was used to generate pharmacophore models, which are important for their inhibitory activities against CA. Furthermore, the association of CA inhibitors with the binding pocket of receptor was validated by a comparative molecular docking investigation, confirming their inhibitory efficacy. The pharmacophore predicted by the 3D QSAR analysis could be a viable scaffold for the identification of new CA inhibitors as anticancer agents. The virtual screening investigation was carried out using the ZINC database’s pharmacophore [18]. Earlier findings suggested that acetazolamide drug can be taken as reference to produce different hypothetical compounds followed by their docking studies against CA enzyme target. After that, the best compounds were chosen by combining it with ZINC-screened compounds [19]. The hypothetic compounds can also be used for synthesis, as well as in vivo and in vitro research. An enzymatic assay can be used to determine the inhibitors’ target specificity. Molecular docking studies could assist in identifying the inhibitors’ orientation and binding interactions with the enzyme’s active site. To evaluate drug likeness properties, the ADME property and oral toxicity were predicted. As a result, we expect this effort to provide some interesting molecules for the treatment of oxidative stress linked to a variety of pathological complications [20].

## 2. Materials and Methods

### 2.1. Collection of Data Set

**Figure 1:**
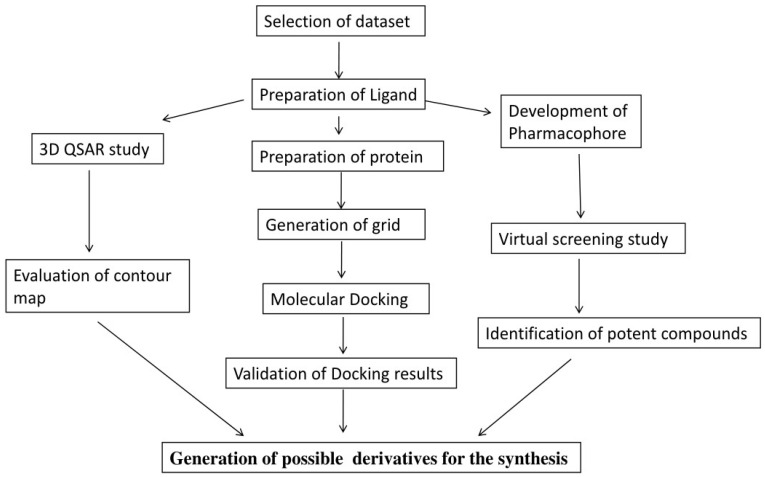
The schematic workflow of 3D QSAR, pharmacophore model, docking, and virtual screening performed in the present study.

Dataset of 27 ligands were taken in the present study [21]. All of the ligands’ structures were drawn in ChemDraw Ultra 12.0 software [22] and saved in ‘.mol’ format. The biological activity was reported in literature in terms of IC_50_ values (in µM), which were converted to pIC_50_ for QSAR studies, as shown in Table 1. pIC50 is represented as the negative logarithmic value of IC50, so first the IC50 value was converted into molar concentration, then into pIC50 value. To generate the predicted pIC_50_ values for 3D-QSAR analysis, the complete dataset was divided in 7:3 training and test sets. The complete process of the present study is depicted in Figure 1.

### 2.2. Preparation of Ligands

**Figure 2:**
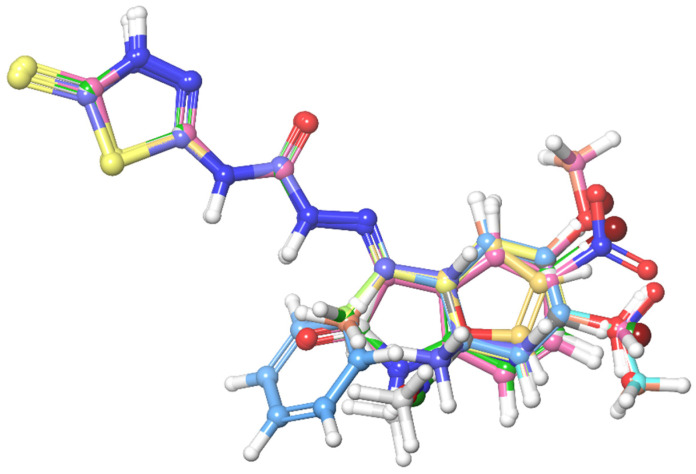
Molecular alignment of all the compounds taken in the QSAR study.

The LigPrep [23] module was used to prepare the ligand via Maestro, which was specifically configured to provide input structures for the Glide and PHASE modules. Clean up wizard can process one ligand per second at a time, effectively converting large datasets from 2D to 3D structures, as well as essential steps in pharmacophore development and docking studies, utilising unique algorithms. To discover the optimal alignment and common features for 3D QSAR model generation, the molecules were aligned based on their most common core structure, as shown in Figure 2.

### 2.3. Pharmacophore Mapping

**Figure 3:**
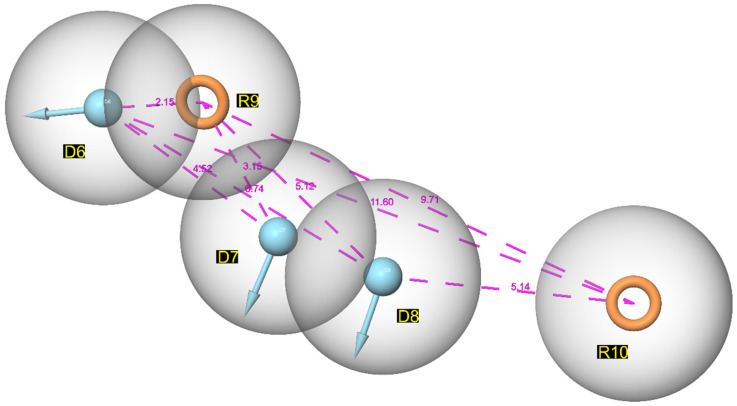
Common pharmacophoric features of generated module (DDDRR_1) with hydrogen bond donor (D) and ring aromaticity (R).

Pharmacophore mapping is the process of defining and placing unique pharmacophoric features as well as using alignment algorithms to overlay 3D conformations [24]. The PHASE module of the Schrodinger Maestro software was used to carry out the pharmacophore mapping study. There are various features present in developed pharmacophore hypothesis, such as hydrogen bond acceptors (A), hydrogen bond donor (D), aromatic ring (R), and a hydrophobic (H) group. The top ranked hypothesis consists of two unique pharmacophoric features based on structural requirements for biological activity: hydrogen bond donor (D) and ring aromaticity (R). As shown in Figure 3, each feature in this pharmacophoric map signifies a common particular portion associated with the selected compounds.

### 2.4. Pharmacophore Hypothesis Generation

Using the PHASE module’s “Developing a pharmacophore model” [25,26], 20 hypotheses were created to explain how active molecules bind to receptors with a box size of 1 Å and a minimum inter site distance of 2 Å. A pharmacophore site is a characteristic feature of a conformation that has been mapped to a specific location. Common pharmacophoric features were identified from a set of variants—feature types that identify a putative pharmacophore. Using a scoring function as a survival score, the common pharmacophore hypotheses were investigated. The site, vector, volume, selectivity scores, and number of matches were calculated for each of the generated hypotheses. The PHASE module has six in-built pharmacophore features, including hydrogen bond acceptor (A), hydrogen bond donor (D), hydrophobic group (H), ring aromaticity (R), positively ionizable (P), and negatively ionizable (N) groups (Supplementary File; Table S1).

### 2.5. An Atom Based 3D-QSAR

Schrodinger Maestro software was used to build an atom-based 3D-QSAR model using a collection of aligned ligands in order to predict activities for additional compounds [27]. The training set consisted of 70% of the dataset’s compounds, while the test set consisted of 30%. The compounds were clustered using a PLS factor of 4. The Atom type fraction segment demonstrated the fraction owing to each atom type in the QSAR model for each number of PLS factors included in the model. Furthermore, actual activity versus predicted activity for compounds in the training as well as the test sets were plotted to form a scatter plot.

### 2.6. Virtual Screening

The virtual screening study was carried out using the ZINC data base, and the AAHRR 1 hypothesis was employed to screen ZINC compounds using the Lipinski rule of 5 (Supplementary File; Table S2) [28]. Using different filters, a total of 3568 molecules were screened. The GLIDE module of Schrodinger was used to screen these compounds using different docking methodologies. The Swiss Target Prediction tool was used to predict the target of these selected compounds. This tool predicts all the available targets of the molecule. Different targets were identified in the current investigation, with CA being the most important one for the particular molecule. 

### 2.7. Molecular Docking

The docking studies were carried out using the Schrodinger Maestro software’s Glide module [29]. The score function in the software was used to rank and group distinct possible adduct structures generated by molecular docking [30] (Supplementary File; Table S3). Based on the binding properties of the ligand and target, it predicts the three-dimensional structure of any complex. The method of predicting ligand conformation and orientation (or posing) within a specific binding site is referred to as docking. In Maestro wizard, the protein structure was pre-processed using the “protein preparation wizard”. Hydrogen atoms and certain essential bonds were introduced to the missing site of the protein molecule by automatically generating states and refinement steps phases of the module. After the optimization process, receptor grid generation was processed and docking scores were analysed with different docked ligand conformations [31,32].

### 2.8. ADME Properties Prediction

The drug-like activity of the ligand molecule was predicted using the Schrodinger software’s QikProp module [33]. It predicts both physicochemical descriptors and pharmacokinetic properties with the objective of increasing the success rate of compounds for further development. The ADME study suggests that drug-like properties of ligand molecules were validated using Lipinski’s rule of five [34,35]. The rule states that the molecular weight <500, Log P_o/w_ <5, hydrogen bond donor ≤5, and hydrogen bond acceptor ≤10. The compounds that meet these criteria are classified as drug-like compounds (Supplementary File; Tables S4 and S5).

## 3. Results and Discussion

### 3.1. Pharmacophore Mapping: Selection of the Best Pharmacophore Hypothesis

All the selected compounds from the database were used to generate pharmacophoric hypothesis or the minimum features of the compound required to bind with receptor. Among 20 pharmacophore hypotheses, we screened best pharmacophoric hypotheses on the basis of various scores described in Table 2. Three hydrogen bond donors (D) and two ring aromaticity (R) were identified as common pharmacophore features. DDDRR 1 and DDDRR 2 were found to be the best among the 20 hypotheses generated (Supplementary File; Table S1) by the PHASE module, based on a scoring function of each parameter value listed in Table 2 [36]. The “survival” scoring (S) function was responsible for determination of features from described models and to rank all hypotheses. The scoring algorithm involves selectivity, number of ligands matched, relative conformational energy, and activity. However, the models should also differentiate between inactive and active molecules. If an inactive molecule scores well, the hypothesis could be invalid as it does not discriminate between active and inactive molecules. For this reason, adjusted survival score S_I was calculated by subtracting the inactive score from the survival score. 

### 3.2. Selection of Atom Based QSAR Model

The QSAR findings for the hypothesis are shown in the statistical table obtained for the training and test set molecules. Several statistical parameters were employed to examine the robustness of the QSAR model, including SD, R^2^, F, P, RMSE, Q^2^, and Pearson-R [37]. High R^2^ (higher than 0.6), Q^2^ (greater than 0.5), Pearson-R (greater than 0.5), and F values characterize a good QSAR model. All the four models generated by the module are shown in Table 3. Based on the above information, the fourth model was chosen as the best QSAR model due to higher Q^2^ and R^2^ values as 0.7888 and 0.8757, respectively.

**Figure 4:**
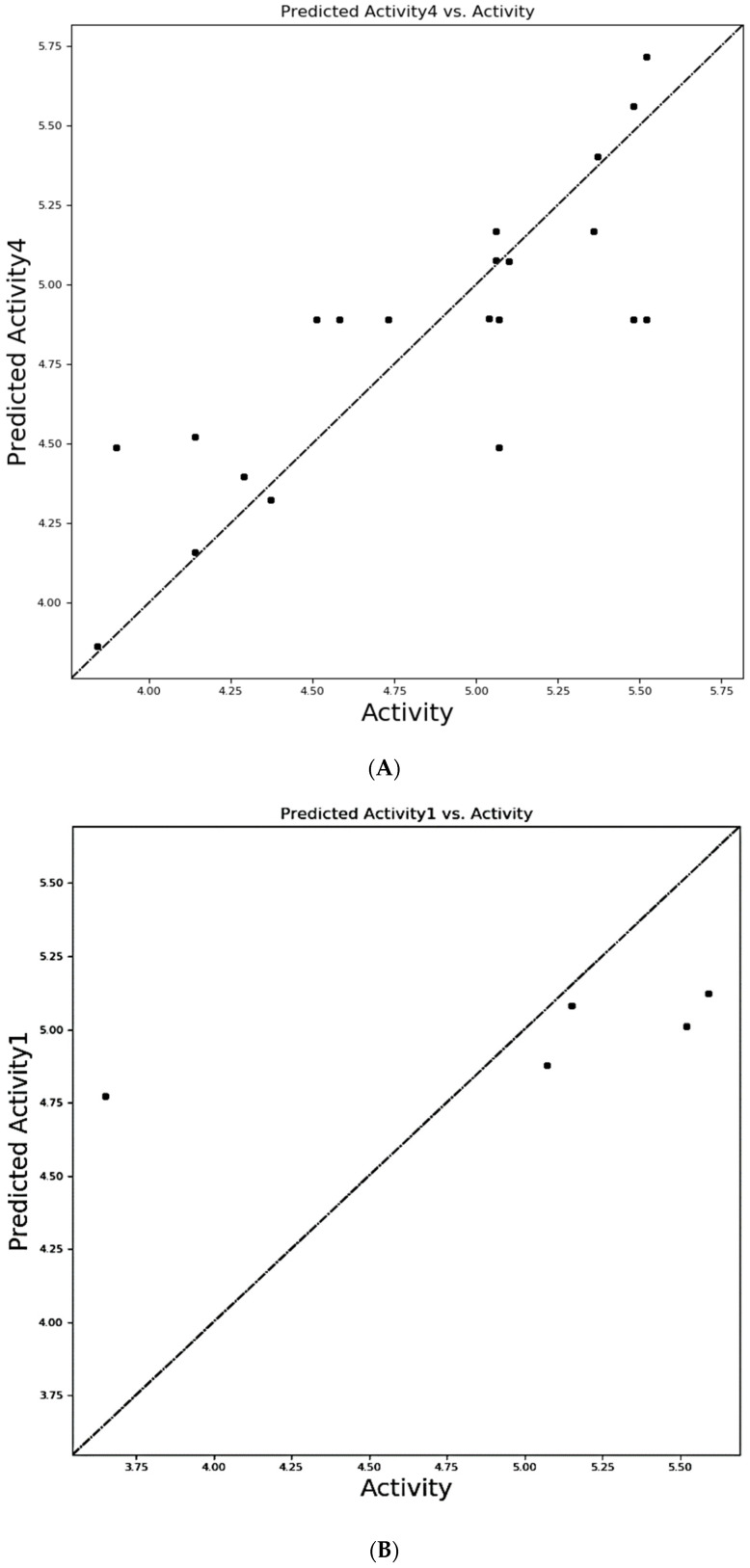
Scattered plot of experimental vs. predicted activity of training set (**A**) and test set (**B**) molecules, where the filled circles represent the training and test set compounds, respectively.

To demonstrate the uniform distribution of training set molecules over the straight line passing through the origin (0, 0), a scattered plot was drawn between experimental activity and predicted activity of training and test set compounds (Figure 4).

### 3.3. Evaluation of Contour Map

**Figure 5:**
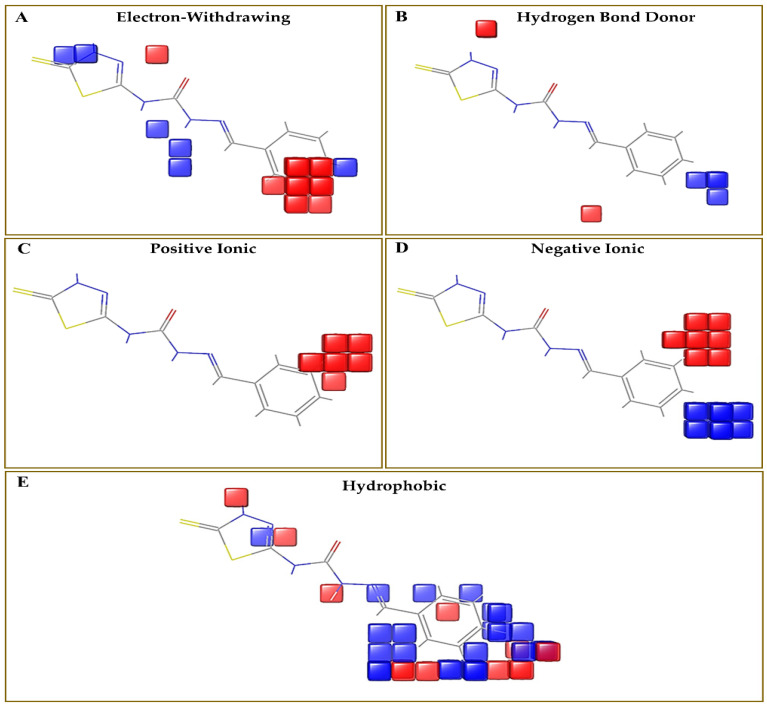
3D-QSAR model (atom-based) consisting of (**A**) electron-withdrawing, (**B**) hydrogen bond donor, (**C**) positive ionic, (**D**) negative ionic, (**E**) hydrophobic groups, where the blue cubes showed positive coefficients and the red cubes showed negative coefficients.

The effects of different substituents on biological activity were determined by the study of contour maps, which are shown in Figure 5A–E. It also examines the variation of substituents and their biological activity. The contour map is represented by color coding, including different substituents on the core moiety. This color coding has a specific indication of the substituent and is useful in the expansion of novel compounds as CA inhibitors. The blue color showed enhanced activity, whereas the red color indicated decrease in activity. The electron-withdrawing group substitution on the phenyl ring showed a decrease in activity. Hydrogen bond donor group substitution on phenyl ring with para substitution showed an increase in activity. Hydrophobic substitution at the ortho position displayed good activity. Negative ionic group showed good activity at the para position and deceased activity in the meta position. The positive ionic group substitution in the phenyl ring showed decrease in activity. 

### 3.4. Molecular Docking Analysis

**Figure 6:**
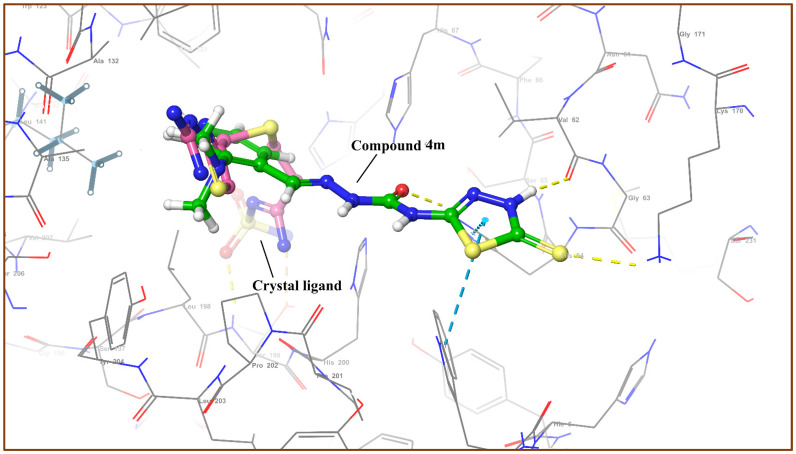
3D interactions of compound **4m** and crystal ligand with different amino acids His64, Val62 at the binding site of receptor 6g3v.

**Figure 7:**
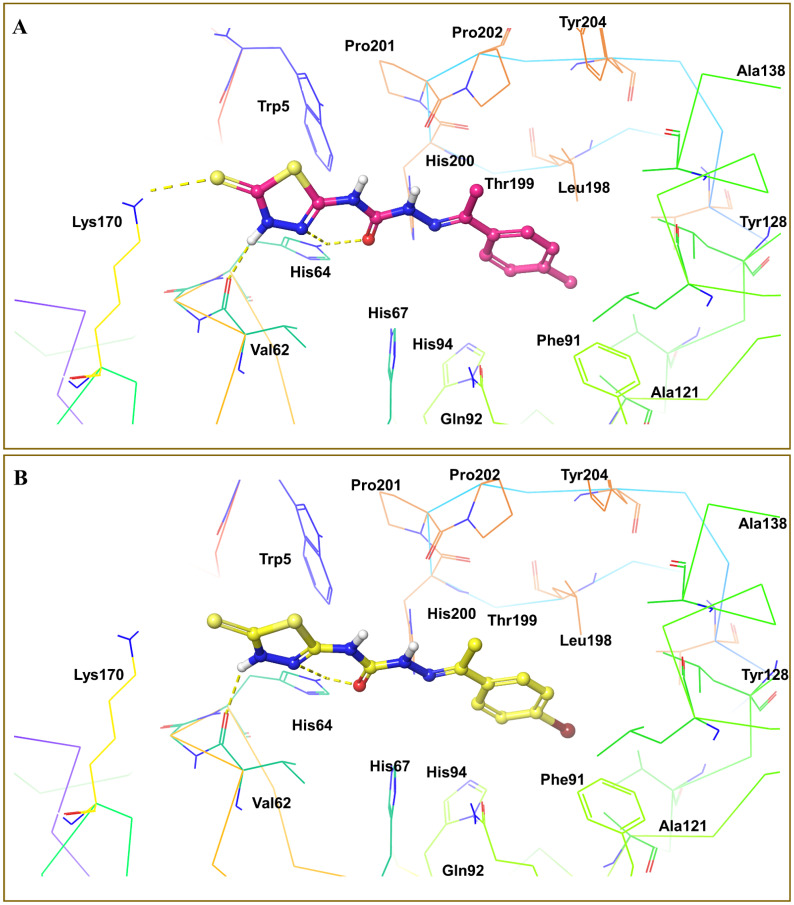
(**A**) 3D interactions of compound **4o** with different amino acids His64, Val62, Lys170 at the binding site of receptor 6g3v. (**B**) 3D interactions of compound **4s** with different amino acids His64, and Val62 at the binding site of receptor 6g3v.

The Glide module was utilized to conduct molecular docking between the potent derivatives and the target protein [38,39]. The potent analogues **4m**, **4o**, **4s**, **4p**, and **5b** demonstrated the highest SP docking scores of −5.217, −4.866, −4.729, −4.641, and −4.635 respectively, when interacting with amino acids of the target protein. The docking scores of compounds in the dataset are shown in Table 4. The purple arrows indicate hydrogen bond interactions, whereas the green arrows indicate π-π-stacking interactions, as seen in Figure 6 for compound **4m**, Figure 7A for compound **4o**, and Figure 7B for compound **4s** [40,41].

### 3.5. Virtual Screening

**Figure 8:**
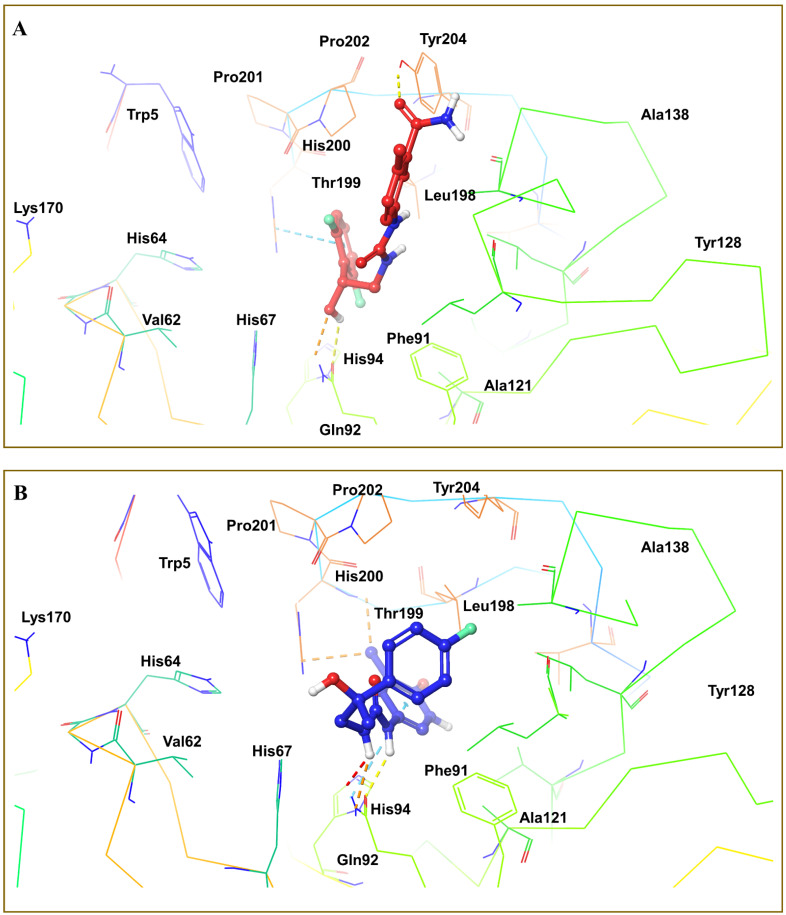
(**A**) 3D interactions of compound ZINC77699643 with different amino acids—Pro201 and Thr199—at the binding site of receptor 6g3v. (**B**) 3D interactions of compound ZINC89275054 with different amino acids—His94, Gln92, and Thr199—at the binding site of receptor 6g3v.

**Figure 9:**
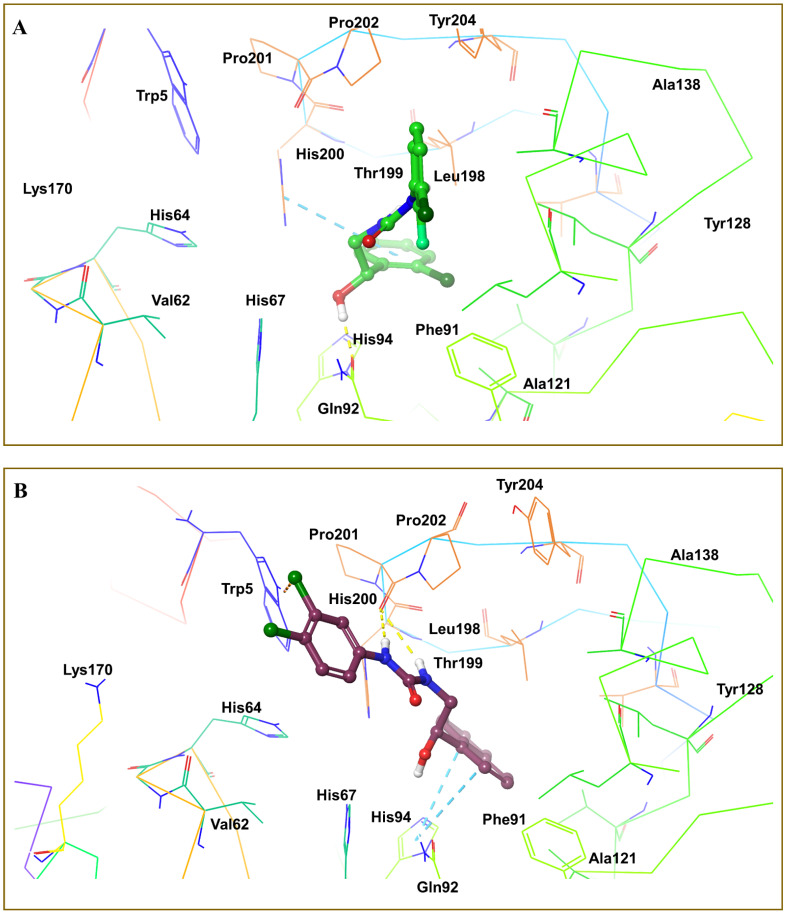
(**A**) 3D interactions of compound ZINC77671412 with different amino acids—His200 and Gln92—at the binding site of receptor 6g3v. (**B**) 3D interactions of compound ZINC70762033 with different amino acids—His200, His94, Gln92, and Thr199—at the binding site of receptor 6g3v.

The molecular docking methodologies such as HTVS, SP, and XP were applied to screen the compounds from the ZINC database. Each step screened 20% of the highest-scoring compounds with high docking scores. The top compounds, ZINC77699643, ZINC89275054, ZINC77671412, and ZINC70762033, were selected with xp docking scores of −6.178, −5.743, −5.561, and −5.535, respectively, out of 333 compounds screened using the SP docking approach. These compounds were chosen as the final ZINC compounds for further investigation and assessed using MMGBSA to calculate binding interaction energy. The compound ZINC77699643 interacted with Pro201 and Thr199 amino acid residues, as shown in Figure 8A. These interactions demonstrated the significance of CA activity. The compound ZINC89275054 showed binding interactions with amino acid residues His94, Gln92, and Thr199 in the same cavity, as shown by a crystal ligand (Figure 8B). The compound ZINC77671412 demonstrated binding interactions with several amino acids at the receptor’s binding region, including His200 and Gln92, which are critical for activity (Figure 9A). The compound ZINC70762033 showed binding interaction with different amino acids such as His200, His94, Gln92, and Thr199 at the binding site of receptor (Figure 9B).

### 3.6. ADME Properties Prediction

The ADME properties of all the compounds from the dataset were predicted through the QikProp module of Schrodinger; the results are given in Table 5. The software predicted the compounds’ physicochemical properties, lipophilicity, drug-like behaviour, water solubility, permeability through BBB, pharmacokinetics, and synthetic accessibility [42,43]. All compounds have a molecular weight ranging from 259.34 to 399.24, number of hydrogen bond acceptors ranging from 4.5 to 7.0, and number of hydrogen bond donors ranging from 3 to 4. All the predicted ADME properties satisfied the Lipinski’s rule of five for all the compounds.

### 3.7. MMGBSA-Based Rescoring 

The MMGBSA-based rescoring method was used for calculation of binding free energy for ligands and ZINC hit compounds ZINC77699643, ZINC89275054, ZINC77671412, and ZINC70762033 (complex with PDB ID: 6g3v), which provided very high binding free energy, as dG bind −69.8, −84.17, −67.67, −82.2 kcal/mol, respectively (Table 4)**.**

## 4. Optimization of Novel Ligands

**Figure 10:**
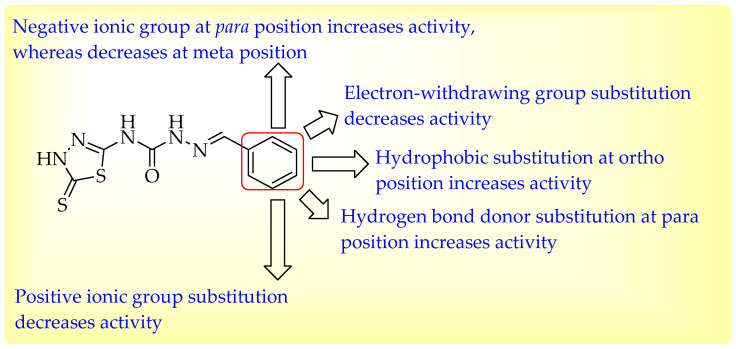
Optimization of ligands for the development of novel thiadiazole derivatives.

Optimization of ligands revealed that the substitution of the hydrogen bond donor group on the phenyl ring at the para position, hydrophobic group at the ortho position, and negative ionic group at the para position showed increase in activity. However, substitution of the negative ionic group at the meta position of the phenyl ring, and positive ionic group substitution on the phenyl ring displayed a decrease in activity (Figure 10). 

## 5. Conclusions

The computational design of 1,3,4-thiadiazole derivatives as potential CA inhibitors was discussed in this study. The PHASE module (Schrodinger) was used to investigate the pharmacophore model on 27 compounds taken from a dataset. The compound **4m** [(2-(2-(dimethylamino)benzylidene)-*N*-(5-thioxo-4,5-dihydro-1,3,4-thiadiazol-2-yl)hydrazine-1-carboxamide)] showed the best SP docking score of −5.217. The predicted ADME properties revealed that all the compounds followed the Lipinski’s rule of five, indicating their drug-like potential. Optimization of ligands can help to generate novel thiadiazole derivatives by using the response of a contour map. The generated contour map showed the substitution of the electron-withdrawing group on the phenyl ring responsible for decrease in CA activity. However, the hydrophobic group substitution at the ortho position, and hydrogen bond donor substitution at the para position of the phenyl ring showed an increase in CA activity. These results may aid in the development of novel thiadiazole compounds as CA inhibitors, which might have a major influence on the treatment of oxidative stress induced by low oxygen pressure at high altitudes.

## Figures and Tables

**Table 1:** Compounds taken in QSAR study with CA inhibitory activity (IC_50_ and pIC_50_ value) [21].

S. No.	Compounds	Structures	IC_50_ Value (µM)	pIC_50_ Value
1	**4a**	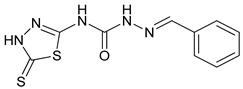	3	5.52
2	**4b**	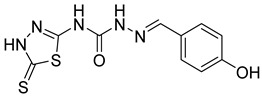	8.03	5.52
3	**4c**	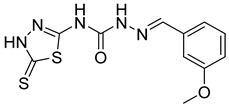	8.54	5.07
4	**4d**	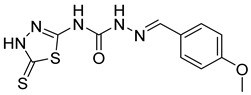	26.16	5.07
5	**4e**	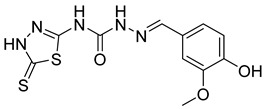	51.63	5.52
6	**4f**	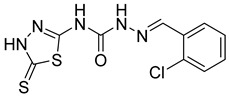	3.28	5.10
7	**4g**	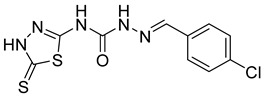	18.74	5.07
8	**4h**	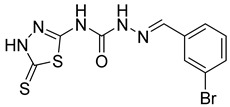	71.62	4.58
9	**4i**	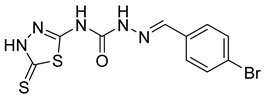	2.55	4.29
10	**4j**	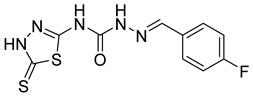	30.77	5.48
11	**4k**	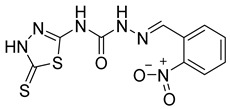	222.82	4.73
12	**4l**	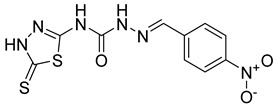	7.11	4.14
13	**4m**	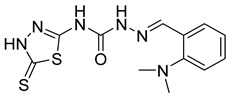	125	5.59
14	**4n**	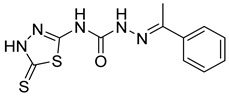	9.09	4.51
15	**4o**	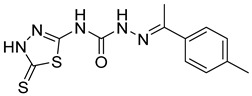	4.32	3.65
16	**4p**	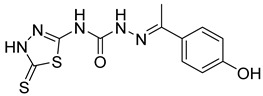	3.34	5.15
17	**4q**	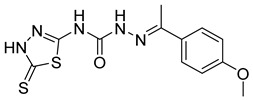	4.31	3.90
18	**4r**	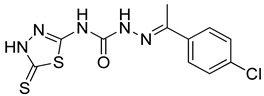	144.07	5.04
19	**4s**	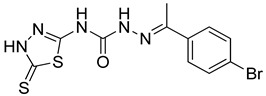	42.25	5.36
20	**4t**	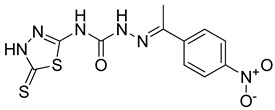	8.62	5.48
21	**4u**	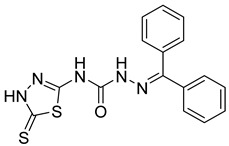	4.34	5.37
22	**4v**	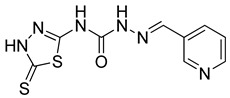	8.64	3.84
23	**4w**	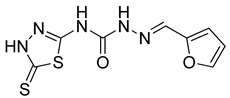	73.25	4.37
24	**4x**	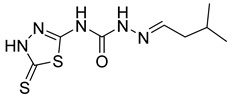	8.53	5.06
25	**5a**	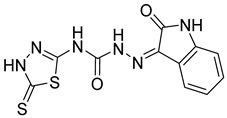	7.89	5.36
26	**5b**	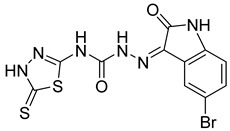	3.71	5.06
27	**5c**	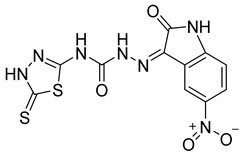	5.95	4.14

**Table 2:** Pharmacophore hypothesis with ranking on the basis of survival scores.

HypoID	Survival	Site	Vector	Volume	Select	Matches	Inactive	Adjusted	BEDROC
DDDRR_1	5.4403	1	1	0.9301	1.9083	4	2.7979	2.6424	1
DDDRR_2	5.4403	1	1	0.9301	1.9083	4	2.7979	2.6424	1
DDDRR_3	5.4344	0.9999	1	0.9303	1.9021	4	2.8723	2.562	1
DDDRR_4	5.4336	1	1	0.9306	1.901	4	2.5881	2.8455	1
DDDRR_5	5.4233	1	1	0.9307	1.8906	4	2.5503	2.873	1
ADDRR_1	5.2355	1	1	0.93	1.7036	4	2.8242	2.4114	1
ADDRR_2	5.2177	1	1	0.9301	1.6857	4	2.898	2.3197	1
ADDRR_3	5.2028	1	1	0.8972	1.7036	4	2.7787	2.4241	1
ADDRR_4	5.1937	1	1	0.8979	1.6937	4	2.427	2.7667	1
ADDRR_5	5.1834	0.9999	1	0.9301	1.6513	4	2.8282	2.3553	1
DDRR_1	4.9585	1	1	0.9301	1.4264	4	2.8214	2.137	1
DDRR_2	4.9585	1	1	0.9301	1.4264	4	2.8214	2.137	1
DDRR_3	4.9527	1	1	0.9303	1.4204	4	2.9038	2.0489	1
DDRR_4	4.9489	1	1	0.9301	1.4168	4	2.7901	2.1588	1
DDRR_5	4.9488	1	1	0.9306	1.4162	4	2.6459	2.3029	1
DDRR_6	4.9475	1	1	0.9303	1.4153	4	2.8664	2.0812	1
DDRR_7	4.9451	0.9999	1	0.9303	1.4129	4	2.8672	2.0779	1
DDRR_8	4.9444	1	1	0.9307	1.4117	4	2.6223	2.3221	1
DDRR_9	4.9418	1	1	0.9306	1.4092	4	2.5853	2.3564	1
DDRR_10	4.9388	1	1	0.9307	1.4061	4	2.5917	2.3471	1

**Table 3:** Atom-based QSAR statistics with important values like R^2, Q^2 and R^2 CV.

# Factors	SD	R^2	R^2 CV	R^2 Scramble	F	P	RMSE	Q^2	Pearson-r
1	0.4137	0.474	0.043	0.3174	17.1	0.00056	0.6	0.2794	0.8494
2	0.3767	0.5867	0.0764	0.4491	12.8	0.000352	0.51	0.4802	0.8547
3	0.3599	0.8438	0.8096	0.549	10.2	0.000438	0.47	0.7448	0.8023
4	0.3539	0.8757	0.8277	0.5943	58.3	0.000784	0.5	0.7888	0.7495

**Table 4:** Docking scores and MMGBSA-based rescores of compounds taken for QSAR and ZINC database.

S. No.	Compound	Docking Score	MMGBSA dG Bind (XPcomplex) kcal/mol
1	**4m**	−5.217	−72.8
2	**4o**	−4.866	−80.17
3	**4s**	−4.729	−74.67
4	**4p**	−4.641	−83.2
5	**5b**	−4.635	−76.8
6	ZINC77699643	−6.178	−69.8
7	ZINC89275054	−5.743	−84.17
8	ZINC77671412	−5.561	−67.67
9	ZINC70762033	−5.535	−82.2

**Table 5:** Result of ADME properties of compounds taken for QSAR analysis.

Compound	CNS	MW (<500)	Dipole	HBD (<5)	HBA (<10)	QPlogPo/w (≤5)	Rule of Five (≤1)	Rule of Three
**4a**	−1	279.334	8.68	3	5	1.331	0	0
**4b**	−2	295.333	7.564	4	5.75	0.601	0	0
**4c**	−2	309.36	7.389	3	5.75	1.447	0	0
**4d**	−2	309.36	9.912	3	5.75	1.446	0	0
**4e**	−2	325.36	8.614	4	6.5	0.754	0	0
**4f**	−1	313.779	7.85	3	5	1.744	0	0
**4g**	−1	313.779	7.342	3	5	1.805	0	0
**4h**	−1	358.23	9.087	3	5	1.878	0	0
**4i**	−1	358.23	7.462	3	5	1.878	0	0
**4j**	−1	297.325	7.295	3	5	1.558	0	0
**4k**	−2	324.332	7.092	3	6	0.713	0	0
**4l**	−2	324.332	6.573	3	6	0.662	0	0
**4m**	−2	322.402	8.929	3	6	1.811	0	0
**4n**	−1	293.361	9.244	3	4.5	1.753	0	0
**4o**	−1	307.388	9.628	3	4.5	2.037	0	0
**4p**	−2	309.36	10.476	4	5.25	1.009	0	0
**4q**	−1	323.387	10.496	3	5.25	1.907	0	0
**4r**	−1	327.806	7.953	3	4.5	2.231	0	0
**4s**	−1	372.257	8.067	3	4.5	2.305	0	0
**4t**	−2	338.358	7.204	3	5.5	1.079	0	0
**4u**	−1	355.432	9.928	3	4.5	2.994	0	0
**4v**	−2	280.322	6.159	3	6.5	0.678	0	0
**4w**	−1	269.296	8.4	3	5.5	0.734	0	0
**4x**	−2	259.344	9.12	3	5	0.822	0	0
**5a**	−2	320.343	10.649	3	6	0.683	0	0
**5b**	−2	399.239	10.195	3	6	1.227	0	0
**5c**	−2	365.341	10.978	3	7	0.016	0	0

## Data Availability

The authors confirm that the data supporting the study’s findings are included in the article and its Supplementary Information.

## Supplementary Materials

The following supporting information can be downloaded at:https://www.mdpi.com/article/10.3390/cimb44030068/s1, Table S1: The pharmacophore study carried out for the dataset; Table S2: The HTVS docking study carried out for the ZINC compounds; Table S3: The docking study carried out for the dataset; Table S4: The QIKPROP study carried out the ZINC database; Table S5: The ADME study carried out for the database.
