# Supplementary material for: An Insight into the Structural Requirements and Pharmacophore Identification of Carbonic Anhydrase Inhibitors to Combat Oxidative Stress at High Altitudes: An In-Silico Approach
Source: Curr Issues Mol Biol. 2022 Feb 23;44(3):1027–45. doi: 10.3390/cimb44030068 (PMC8947748; doi:10.3390/cimb44030068)
Supplement: Supplementary file 1 [file cimb-44-00068-s001.zip › cimb-1573271-supplementary.pdf]

current issues in molecular biology

Article

An Insight into the Structural Requirements and Pharmacophore Identification of Carbonic Anhydrase Inhibitors to combat oxidative stress at High Altitudes: An *In-Silico* Approach

Amena Ali [1,2,] *, Abuzer Ali [3,], Musarrat Husain Warsi [4], Mohammad Akhlaquer Rahman [4], Mohamed Jawed Ahsan [5] and Faizul Azam [6]

1 High Altitude Research Center, Taif University, P.O. Box 11099, Taif 21944, Saudi Arabia; amrathore@tu.edu.sa
2 Department of Pharmaceutical Chemistry, College of Pharmacy, Taif University, P.O. Box 11099, Taif 21944, Saudi Arabia; amrathore@tu.edu.sa
3 Department of Pharmacognosy, College of Pharmacy, Taif University, P.O. Box 11099, Taif 21944, Saudi Arabia; abuali@tu.edu.sa
4 Department of Pharmaceutics and Industrial Pharmacy, College of Pharmacy, Taif University, P.O. Box 11099, Taif 21944, Saudi Arabia; mvarsi@tu.edu.sa (M.H.W.); mrahman@tu.edu.sa ((M.A.R.)
5 Department of Pharmaceutical Chemistry, Maharishi Arvind College of Pharmacy, Ambabari Circle, Jaipur, Rajasthan 302 039, India; jawedpharma@gmail.com
6 Department of Pharmaceutical Chemistry & Pharmacognosy, Unaizah College of Pharmacy, Qassim University, P.O. Box 5888, Unaizah 51911, Saudi Arabia; f.azam@qu.edu.sa
* Correspondence: amrathore@tu.edu.sa

Table S1. The pharmacophore study carried out for the dataset

HypoID	Survival	Site	Vector	Volume	Select	Matches	Inactive	Adjusted	BEDROC	RefLig
DDDRR_1	5.4403	1	1	0.9301	1.9083	4	2.7979	2.6424	1	mol_2
DDDRR_2	5.4403	1	1	0.9301	1.9083	4	2.7979	2.6424	1	mol_2
DDDRR_3	5.4344	0.9999	1	0.9303	1.9021	4	2.8723	2.562	1	mol_2
DDDRR_4	5.4336	1	1	0.9306	1.901	4	2.5881	2.8455	1	mol_2
DDDRR_5	5.4233	1	1	0.9307	1.8906	4	2.5503	2.873	1	mol_2
ADDRR_1	5.2355	1	1	0.93	1.7036	4	2.8242	2.4114	1	mol_2
ADDRR_2	5.2177	1	1	0.9301	1.6857	4	2.898	2.3197	1	mol_2
ADDRR_3	5.2028	1	1	0.8972	1.7036	4	2.7787	2.4241	1	mol_5
ADDRR_4	5.1937	1	1	0.8979	1.6937	4	2.427	2.7667	1	mol_5
ADDRR_5	5.1834	0.9999	1	0.9301	1.6513	4	2.8282	2.3553	1	mol_2
DDRR_1	4.9585	1	1	0.9301	1.4264	4	2.8214	2.137	1	mol_2
DDRR_2	4.9585	1	1	0.9301	1.4264	4	2.8214	2.137	1	mol_2
DDRR_3	4.9527	1	1	0.9303	1.4204	4	2.9038	2.0489	1	mol_2
DDRR_4	4.9489	1	1	0.9301	1.4168	4	2.7901	2.1588	1	mol_2
DDRR_5	4.9488	1	1	0.9306	1.4162	4	2.6459	2.3029	1	mol_2
DDRR_6	4.9475	1	1	0.9303	1.4153	4	2.8664	2.0812	1	mol_2
DDRR_7	4.9451	0.9999	1	0.9303	1.4129	4	2.8672	2.0779	1	mol_2

DDRR_8	4.9444	1	1	0.9307	1.4117	4	2.6223	2.3221	1	mol_2
DDRR_9	4.9418	1	1	0.9306	1.4092	4	2.5853	2.3564	1	mol_2
DDRR_10	4.9388	1	1	0.9307	1.4061	4	2.5917	2.3471	1	mol_2

Table S2. The HTVS docking study carried out for the ZINC compounds

**s_lp_Variant**	**r_i_glide_energy**	**r_sd_rmsd**	**r_i_docking_score**	**r_i_glide_erotb**	**s_lp_Force_Field**
ZINC70762033-1	-38.59	0.57	-5.01	0.55	OPLS_2005
ZINC70762031-2	-38.59	0.64	-5.01	0.55	OPLS_2005
ZINC40097700-1	-38.59	0.67	-4.99	0.77	OPLS_2005
ZINC90623072-2	-38.69	0.56	-4.99	0.93	OPLS_2005
ZINC40097521-1	-22.73	0.54	-4.89	0.64	OPLS_2005
ZINC77699643-2	-38.11	0.55	-4.87	0.78	OPLS_2005
ZINC40097670-2	-36.94	0.57	-4.87	0.64	OPLS_2005
ZINC77699643-1	-38.71	0.55	-4.84	0.78	OPLS_2005
ZINC89999382-1	-37.95	0.60	-4.81	0.71	OPLS_2005
ZINC71926558-1	-30.90	0.59	-4.81	0.90	OPLS_2005
ZINC72277793-1	-32.99	0.33	-4.79	1.06	OPLS_2005
ZINC93366947-1	-36.89	0.30	-4.73	0.68	OPLS_2005
ZINC90626422-1	-35.75	0.51	-4.73	0.95	OPLS_2005
ZINC90745935-2	-35.87	0.62	-4.71	0.77	OPLS_2005
ZINC83312205-2	-35.82	0.53	-4.71	0.81	OPLS_2005
ZINC78644559-3	-34.19	0.69	-4.67	0.72	OPLS_2005
ZINC90480109-1	-38.89	0.58	-4.66	0.80	OPLS_2005
ZINC77507712-2	-37.88	0.56	-4.66	0.99	OPLS_2005
ZINC70762033-2	-39.05	0.57	-4.62	0.55	OPLS_2005
ZINC70762031-1	-39.05	0.64	-4.62	0.55	OPLS_2005
ZINC75978306-1	-26.05	0.54	-4.59	0.59	OPLS_2005
ZINC56658160-2	-37.12	0.59	-4.56	1.10	OPLS_2005

Table S3. The docking study carried out for dataset

**Molecule**	**glide lig-num**	**glide rotatable bonds**	**docking score**	**glide ligand efficiency**	**glide ligand efficiency sa**	**glide ligand efficiency ln**	**glide gscore**	**glide lipo**	**glide hbond**
ZINC92094001	13	6	-5.217	-0.248	-0.685	-1.29	-5.217	-1.519	-0.563
ZINC92094001	15	5	-4.866	-0.243	-0.66	-1.218	-4.866	-1.361	-0.596
ZINC72277793	19	5	-4.729	-0.236	-0.642	-1.183	-4.729	-1.354	-0.517

ZINC72277793	16	6	-4.641	-0.232	-0.63	-1.161	-4.641	-0.965	-0.32
ZINC72277793	26	4	-4.635	-0.211	-0.59	-1.133	-4.635	-1.051	-0.596
ZINC72277793	27	5	-4.571	-0.19	-0.549	-1.094	-4.571	-1.039	-0.135
ZINC89580337	2	6	-4.385	-0.231	-0.616	-1.112	-4.385	-0.848	-0.32
ZINC89580337	7	5	-4.309	-0.227	-0.605	-1.093	-4.309	-1.071	-0.166
ZINC55245475	20	6	-4.283	-0.195	-0.545	-1.047	-4.283	-1.329	0
ZINC44254082	18	5	-4.24	-0.212	-0.575	-1.061	-4.24	-1.093	-0.32
ZINC77509952	5	7	-4.163	-0.198	-0.547	-1.029	-4.163	-1.037	0
ZINC77509952	1	5	-4.115	-0.229	-0.599	-1.058	-4.115	-0.968	-0.099
ZINC91628968	17	6	-4.107	-0.196	-0.54	-1.016	-4.107	-1.096	-0.295
ZINC91628968	25	4	-4.09	-0.195	-0.537	-1.011	-4.09	-0.689	-0.111
ZINC21101702	23	5	-4.069	-0.239	-0.615	-1.061	-4.069	-0.771	-0.179
ZINC21101702	12	6	-4.058	-0.193	-0.533	-1.003	-4.058	-0.967	-0.179
ZINC92068025	6	5	-4.044	-0.213	-0.568	-1.025	-4.044	-0.851	-0.08
ZINC92068025	10	5	-3.919	-0.206	-0.55	-0.994	-3.919	-0.681	-0.08
ZINC40097760	21	6	-3.813	-0.159	-0.458	-0.913	-3.813	-1.686	-0.247
ZINC40097760	11	6	-3.778	-0.18	-0.496	-0.934	-3.778	-0.885	-0.006
ZINC81110469	9	5	-3.699	-0.195	-0.519	-0.938	-3.699	-0.899	-0.131
ZINC81110469	8	5	-3.693	-0.194	-0.519	-0.936	-3.693	-0.842	0
ZINC40086414	3	6	-3.608	-0.18	-0.49	-0.903	-3.608	-0.772	0

ZINC40086414	4	6	-3.524	-0.176	-0.478	-0.882	-3.524	-0.898	0
ZINC43644446	22	5	-3.48	-0.193	-0.507	-0.895	-3.48	-0.439	0
ZINC43644446	14	5	-3.464	-0.182	-0.486	-0.878	-3.464	-0.714	0
ZINC92056766	24	6	-3.297	-0.206	-0.519	-0.874	-3.297	-0.884	0

Table S4. The QIKPROP study carried out ZINC database

**Molecule**	**mol_MW**	**Dipole**	**SASA**	**FOSA**	**FISA**	**PISA**	**donorHB**	**acceptHB**	**dip^2/V**	**QPPCaco**
ZINC78402745	272.321	7.175	580.523	155.686	65.598	320.63	2	2	0.053596	1640.57
ZINC92094001	287.364	4.83	591.116	306.052	95.369	189.696	2	4.5	0.022991	833.33
ZINC92094001	287.364	5.105	590.675	305.907	93.174	191.594	2	4.5	0.025696	890.54
ZINC72277793	293.297	3.904	538.26	202.226	126.233	162.772	3	5.2	0.016262	517.41
ZINC72277793	293.297	4.073	566.3	189.468	141.532	188.277	3	5.2	0.01739	306.95
ZINC72277793	293.297	10.466	571.968	189.517	133.627	201.802	3	5.2	0.114168	340.75
ZINC72277793	293.297	8.899	555.374	202.292	119.3	186.753	3	5.2	0.083651	525.21
ZINC89580337	293.322	9.944	573.624	366.293	118.816	88.516	3	4.75	0.101383	507.02
ZINC89580337	293.322	9.946	570.807	362.897	118.685	89.225	3	4.75	0.101747	506.33
ZINC55245475	293.386	4.67	590.565	325.785	85.886	147.65	2	4.5	0.021758	1016.70
ZINC44254082	294.276	7.254	568.857	71.264	65.54	291.176	2	2	0.056247	1644.59
ZINC77509952	297.261	7.116	554.313	136.457	152.07	204.251	3	5.2	0.055255	250.40
ZINC77509952	297.261	7.215	554.927	136.924	151.727	204.306	3	5.2	0.056776	251.03
ZINC91628968	299.372	6.051	596.206	292.279	115.407	188.521	2	3.5	0.035731	502.38
ZINC91628968	299.372	5.343	589.171	290.332	110.532	188.308	2	3.5	0.028085	580.75
ZINC21101702	300.332	8.422	591.71	188.821	61.815	302.605	2	2.75	0.071769	1627.73
ZINC21101702	300.332	8.422	591.71	188.821	61.815	302.605	2	2.75	0.071769	1627.73

ZINC92068025	301.391	5.482	583.937	324.874	81.134	177.929	2	5	0.028706	1328.99
ZINC92068025	301.391	5.305	594.398	324.891	84.632	184.875	2	5	0.02673	1260.46
ZINC40097760	302.348	5.532	615.354	220.952	100.313	263.367	3	3.7	0.029607	828.79
ZINC40097760	302.348	5.539	617.893	221.696	96.29	263.508	3	3.7	0.029636	830.40
ZINC81110469	302.348	4.754	604.316	201.976	49.765	314.343	2	3.7	0.021957	2343.25
ZINC81110469	302.348	4.574	609.242	200.564	53.838	316.603	2	3.7	0.020283	2028.26
ZINC40086414	304.32	4.789	518.766	120.785	97.622	268.989	3	4.45	0.024657	877.04
ZINC40086414	304.32	7.597	572.894	144.074	97.515	310.546	3	4.45	0.059241	841.60
ZINC43644446	304.348	6.495	625.056	278.317	119.222	227.516	3	5.95	0.040361	511.12
ZINC43644446	304.348	8.672	564.107	265.354	114.365	184.389	3	5.95	0.075218	625.79
ZINC92056766	304.409	4.569	607.654	275.15	100.97	187.736	2	5	0.020125	735.12
ZINC92056766	304.409	4.826	607.852	274.837	99.128	190.063	2	5	0.022462	777.75
ZINC75407574	303.421	5.403	596.595	262.52	52.038	233.642	2	4	0.028138	604.02
ZINC75407574	303.421	5.432	600.324	261.657	52.218	238.065	2	4	0.028387	599.55
ZINC75407574	303.421	5.544	595.514	260.302	52.191	234.639	2	4	0.029749	600.09
ZINC75407574	303.421	4.985	608.393	256.615	56.483	246.897	2	4	0.023875	517.96
ZINC77507712	304.775	5.885	589.344	133.209	93.958	315.159	3	3.7	0.034936	1035.03
ZINC77507712	304.775	5.459	598.276	131.154	95.212	314.76	3	3.7	0.029814	848.36
ZINC81110527	306.311	6.165	580.926	122.761	89.547	300.247	3	3.7	0.038768	1055.77
ZINC81110527	306.311	6.165	580.926	122.761	89.547	300.247	3	3.7	0.03877	1055.77
ZINC93277934	306.367	7.037	612.384	351.172	139.771	121.441	3	6.2	0.046957	336.53
ZINC93277934	306.367	7.075	603.977	356.903	127.48	119.594	3	6.2	0.047651	452.93
ZINC89475098	306.425	5.443	571.646	375.813	40.554	112.441	2	3	0.028797	3213.42
ZINC89475098	306.425	9.004	572.672	372.264	46.195	117.391	2	3	0.079221	2772.39
ZINC81110404	308.303	5.14	565.447	153.531	50.097	237.777	2	2	0.027375	2235.82
ZINC75540557	309.315	6.066	587.621	278.559	77.704	146.932	2	4	0.037027	1204.67

ZINC75540557	309.315	7.136	581.435	294.615	72.946	139.343	2	4	0.051457	1350.40
ZINC75540557	309.315	4.172	580.106	294.709	78.56	126.08	2	4	0.017527	1325.14
ZINC75540557	309.315	4.964	591.733	289.711	84.752	141.638	2	4	0.024603	1025.53
ZINC77180771	310.395	4.554	593.829	300.092	80.432	213.306	3	2.75	0.019592	1231.30
ZINC77180771	310.395	6.314	618.395	302.526	75.319	240.55	3	2.75	0.037205	1333.93
ZINC79818756	311.383	6.261	594.481	241.083	107.135	246.263	3	4.25	0.037671	656.08
ZINC79818756	311.383	3.51	571.943	241.415	97.931	232.596	3	4.25	0.012066	965.85
ZINC38548906	312.33	11.95	625.981	165.426	146.685	313.87	2	6	0.137323	264.70
ZINC38548906	312.33	12.128	636.258	169.503	151.75	315.005	2	6	0.140454	218.92
ZINC21102218	312.368	6.002	622.205	284.864	61.814	275.527	2	3.5	0.03431	1629.34
ZINC21102218	312.368	6.002	622.205	284.864	61.814	275.527	2	3.5	0.03431	1629.34
ZINC90626422	312.368	4.922	599.173	204.074	80.101	314.998	3	3.5	0.02359	1196.24
ZINC90626422	312.368	6.303	567.267	207.382	76.851	283.034	3	3.5	0.039397	1525.22
ZINC56347457	311.426	5.727	651.318	295.77	29.396	326.153	2	4	0.028825	1032.29
ZINC56347457	311.426	5.204	656.404	294.559	29.208	332.636	2	4	0.023747	1045.78
ZINC75409812	314.364	5.966	600.371	120.502	126.473	305.01	2	5.5	0.035536	394.13
ZINC93276497	314.386	3.647	620.248	300.049	73.663	246.536	2	3.75	0.012492	1361.33
ZINC93276497	314.386	4.587	623.224	301.85	74.587	246.786	2	3.75	0.019722	1326.13
ZINC78489532	314.386	5.308	657.454	235.413	115.411	306.63	3	5.2	0.025448	544.01
ZINC78489532	314.386	5.64	668.065	241.184	120.165	306.716	3	5.2	0.028496	454.00
ZINC78489532	314.386	5.165	658.326	235.102	116.849	306.375	3	5.2	0.024084	526.09
ZINC78489532	314.386	2.773	658.017	234.479	117.57	305.968	3	5.2	0.006947	512.34
ZINC90623072	316.374	6.797	603.915	129.269	83.898	343.711	3	3.7	0.043981	1107.98
ZINC90623072	316.374	7.22	594.179	125.744	78.964	342.437	3	3.7	0.049963	1252.89
ZINC93263339	316.377	4.51	645.142	300.822	77.891	238.697	2	3	0.018612	1197.65
ZINC93263339	316.377	5.408	631.607	304.863	67.273	231.181	2	3	0.027018	1456.86

ZINC93263339	316.377	3.386	632.899	303.599	67.761	228.413	2	3	0.010596	1437.61
ZINC93263339	316.377	4.459	645.465	301.738	77.519	238.331	2	3	0.018181	1226.53
ZINC43343865	316.42	8.984	616.862	295.435	103.167	171.39	2	4	0.0767	734.82
ZINC43343865	316.42	8.944	615.672	295.239	102.176	171.448	2	4	0.076078	759.01
ZINC05334030	316.789	5.913	645.537	177.441	90.128	306.37	2	3	0.032792	826.30
ZINC48450727	317.362	8.213	630.934	218.838	65.521	298.269	2	3.75	0.063376	1684.30
ZINC56658160	318.347	10.08	592.192	218.907	93.331	231.645	3	3.5	0.098496	941.61
ZINC56658160	318.347	7.936	594.554	218.844	93.49	233.899	3	3.5	0.060962	940.07
ZINC89275054	319.335	8.45	591.117	191.448	152.09	200.538	4	5.25	0.070189	253.61
ZINC89275054	319.335	8.45	591.12	191.449	152.091	200.539	4	5.25	0.070192	253.60
ZINC38935475	320.406	7.617	591.847	305.844	40.869	210.675	2	3.5	0.055782	3065.18
ZINC38935475	320.406	7.485	606.053	312.068	36.709	221.079	2	3.5	0.053568	3481.29
ZINC48290987	321.332	9.577	577.347	302.759	135.635	138.953	3	6.7	0.09093	348.23
ZINC48290987	321.332	9.698	578.826	300.96	138.824	139.042	3	6.7	0.093369	322.50
ZINC58299473	322.409	6.947	651.426	247.912	75.175	328.338	2	3	0.042843	1364.53
ZINC58299473	322.409	7.005	656.179	247.416	76.371	332.393	2	3	0.043452	1275.32
ZINC71926558	322.766	5.734	582.383	89.388	93.112	286.715	3	3.7	0.032769	944.47
ZINC71926558	322.766	5.734	582.9	89.388	93.109	287.236	3	3.7	0.032765	944.52
ZINC78644559	322.766	5.583	566.524	106.546	69.75	272.085	3	3.7	0.031426	1650.07
ZINC78644559	322.766	9.147	571.204	102.378	77.344	273.223	3	3.7	0.084064	1304.48
ZINC78644559	322.766	9.134	577.597	102.237	73.097	283.752	3	3.7	0.083429	1516.29
ZINC78644559	322.766	5.584	565.932	106.566	69.731	271.512	3	3.7	0.03143	1650.51
ZINC54008775	323.317	4.444	595.598	125.364	58.453	285.777	2	3	0.019521	1874.13
ZINC89223521	323.384	7.006	607.357	237.513	103.616	175.512	3	4.25	0.047352	733.60
ZINC89223521	323.384	6.921	593.807	238.183	95.881	168.964	3	4.25	0.046617	869.75
ZINC06276737	324.209	6.989	564.53	79.98	87.108	267.798	3.5	3	0.050451	1195.41

ZINC90475747	324.302	4.697	564.552	104.447	80.359	240.845	3	2.75	0.022529	1239.93
ZINC90475747	324.302	3.84	590.145	106.318	78.099	266.549	3	2.75	0.014817	1265.55
ZINC89999382	324.302	8.822	573.136	106.336	72.495	258.514	3	2.75	0.078874	1432.61
ZINC89999382	324.302	8.821	573.139	106.336	72.497	258.516	3	2.75	0.078868	1432.53
ZINC40525732	324.302	9.971	569.621	142.464	91.528	220.188	3	3.7	0.100739	970.05
ZINC40525732	324.302	5.077	612.299	133.822	105.464	239.019	3	3.7	0.025392	723.61
ZINC93370074	325.369	5.805	641.304	221.479	194.406	225.419	4	5.7	0.031113	101.98
ZINC93370074	325.369	8.596	641.731	224.452	188.714	228.565	4	5.7	0.068287	110.95
ZINC93370074	325.369	4.901	638.498	224.055	190.312	224.132	4	5.7	0.022267	107.60
ZINC93370074	325.369	4.416	636.53	221.025	195.485	220.02	4	5.7	0.018085	101.88
ZINC21101973	326.394	6.331	652.44	327.145	61.76	263.535	2	3.5	0.036164	1633.75
ZINC21101973	326.394	6.249	652.631	327.164	61.816	263.651	2	3.5	0.035229	1628.62
ZINC40088332	326.73	8.368	583.888	44.864	100.067	289.73	3	3.7	0.071921	833.91
ZINC40088332	326.73	5.684	584.02	44.912	100.031	289.767	3	3.7	0.033173	834.96
ZINC85607974	327.339	2.43	584.918	104.323	140.873	339.722	3	4.75	0.005749	331.67
ZINC85607974	327.339	3.513	610.114	106.756	132.127	371.231	3	4.75	0.011802	391.99
ZINC89223120	327.357	1.253	598.093	183.359	132.55	235.24	3	4.25	0.001483	389.21
ZINC89223120	327.357	4.706	567.575	186.725	103.793	236.728	3	4.25	0.021491	712.59
ZINC58126702	327.425	5.078	674.541	330.414	61.516	282.611	2	4.7	0.022154	1805.55
ZINC58126702	327.425	5.935	661.296	350.829	52.552	257.916	2	4.7	0.030498	2183.35
ZINC58126702	327.425	4.756	680.123	344.502	61.502	274.119	2	4.7	0.019263	1797.91
ZINC58126702	327.425	6.295	641.86	357.955	46.665	237.24	2	4.7	0.034519	2680.08
ZINC77415252	328.367	6.012	627.68	289.416	61.819	276.445	2	4.25	0.033989	1631.05
ZINC77415252	328.367	6.012	627.68	289.416	61.819	276.445	2	4.25	0.033989	1631.05
ZINC76111420	328.388	7.861	568.578	132.898	93.331	309.567	2	4.5	0.061532	917.25
ZINC76111420	328.388	3.411	598.196	128.948	103.017	329.7	2	4.5	0.01123	761.43

ZINC04317497	328.757	7.809	594.317	34.435	164.651	323.631	3	4	0.061194	177.52
ZINC84560114	329.373	10.268	619.661	50.465	141.89	371.479	3	5.2	0.101482	293.44
ZINC84560114	329.373	10	594.402	58.758	129.169	354.454	3	5.2	0.097783	438.29
ZINC77700472	327.428	6.51	671.099	325.55	63.629	281.92	2	5.5	0.036511	441.64
ZINC77700472	327.428	6.684	653.074	330.442	55.74	266.892	2	5.5	0.038832	568.11
ZINC77700472	327.428	3.991	668.731	324.902	64.958	278.87	2	5.5	0.013753	429.25
ZINC77700472	327.428	4.857	677.076	324.154	71.235	281.687	2	5.5	0.02019	329.73
ZINC77700472	327.428	4.404	650.137	328.564	57.243	264.33	2	5.5	0.016906	528.40
ZINC77700472	327.428	7.254	673.941	323.503	68.294	282.144	2	5.5	0.045194	353.44
ZINC77700472	327.428	7.109	651.593	327.898	59.226	264.469	2	5.5	0.044024	502.98
ZINC77700472	327.428	7.456	669.669	322.976	69.762	276.931	2	5.5	0.047844	338.70
ZINC40103376	329.785	10.439	636.307	133.895	170.676	267.187	3	5.2	0.101975	172.92
ZINC40103376	329.785	13.449	637.782	136.826	165.103	271.308	3	5.2	0.169261	184.70
ZINC77515036	330.17	5.456	567.09	130.563	156.254	179.175	3	5.2	0.031258	264.74
ZINC77515036	330.17	7.7	570.061	130.502	146.256	192.091	3	5.2	0.061997	305.22
ZINC77514919	330.17	6.995	571.11	132.818	144.749	192.446	3	5.2	0.051079	320.56
ZINC77514919	330.17	7.005	571.219	132.736	144.7	192.559	3	5.2	0.051217	319.09
ZINC78629022	330.404	9.727	614.03	221.3	142.009	206.887	3	6	0.089873	288.33
ZINC78629022	330.404	9.589	619.93	220.761	148.338	207.069	3	6	0.087314	251.85
ZINC70969481	330.813	5.844	614.514	159.497	62.281	321.136	3	2.75	0.031976	1774.23
ZINC70969481	330.813	4.882	636.356	152.614	81.544	330.598	3	2.75	0.022022	1049.75
ZINC78529937	331.321	2.562	564.257	99.048	108.439	278.159	3	4.5	0.006626	731.67
ZINC78529937	331.321	3.971	576.885	93.059	119.167	278.097	3	4.5	0.0158	570.22
ZINC93366947	332.364	7.518	632.842	124.185	165.177	343.479	3	5	0.052907	177.94
ZINC93366947	332.364	7.965	626.029	132.298	158.291	335.441	3	5	0.059656	228.17
ZINC93366947	332.364	5.548	628.941	123.584	166.857	338.501	3	5	0.028919	171.48

ZINC56725163	332.374	5.279	639.855	264.866	94.628	234.86	3	3.5	0.025174	910.83
ZINC56725163	332.374	7.955	636.116	265.476	93.445	231.809	3	3.5	0.057312	943.25
ZINC90479962	332.374	6.653	603.011	272.969	77.448	214.091	3	4.45	0.041133	1299.89
ZINC90479962	332.374	4.829	573.242	238.179	81.671	214.782	3	4.45	0.02231	1158.09
ZINC90479962	332.374	4.83	572.663	238.224	81.679	214.147	3	4.45	0.022244	1157.80
ZINC90479962	332.374	8.824	618.998	277.819	73.441	229.12	3	4.45	0.071532	1509.97
ZINC77485048	334.392	9.222	626.312	328.451	113.09	137.842	2	5.5	0.080901	541.85
ZINC77485048	334.392	9.268	626.432	328.601	113.259	137.882	2	5.5	0.081702	538.55
ZINC76060829	334.42	8.055	647.263	173.975	67.351	405.937	2	3.5	0.056771	1705.24
ZINC76060829	334.42	3.481	639.122	174.178	67.133	397.811	2	3.5	0.010568	1718.83
ZINC75417669	334.432	8.336	671.563	313.145	98.137	227.525	3	4.45	0.061384	795.60
ZINC75417669	334.432	4.165	670.964	311.488	103.334	223.457	3	4.45	0.015333	745.61
ZINC79413526	334.432	6.709	669.013	353.896	45.532	230.582	2	3.5	0.039858	2468.41
ZINC79413526	334.432	6.213	616.862	339.544	44.017	199.31	2	3.5	0.035382	2807.14
ZINC75407592	333.448	4.249	649.669	351.058	56.463	193.698	2	4.75	0.016104	517.06
ZINC75407592	333.448	7.142	661.452	351.138	64.421	197.524	2	4.75	0.045069	381.97
ZINC75407592	333.448	5.255	618.41	349.575	49.705	196.012	2	4.75	0.025087	627.47
ZINC75407592	333.448	7.076	662.775	347.716	65.079	201.524	2	4.75	0.044326	375.94
ZINC90479277	335.377	2.884	636.748	291.034	130.462	168.207	3	4.25	0.00754	416.81
ZINC90479277	335.377	3.166	641.68	302.791	137.297	154.553	3	4.25	0.009008	318.09
ZINC89999987	336.408	12.024	650.12	314.022	148.993	140.189	3	6.45	0.131448	240.69
ZINC89999987	336.408	3.186	623.318	330.785	138.616	126.868	3	6.45	0.009381	346.57
ZINC89999987	336.408	8.884	634.95	331.495	131.666	125.215	3	6.45	0.072473	390.22
ZINC89999987	336.408	6.958	634.805	334.436	124.208	129.333	3	6.45	0.044417	459.24
ZINC89998740	336.793	6.218	589.709	177.203	72.827	230.462	3	2.75	0.037163	1431.51
ZINC89998740	336.793	6.822	561.415	155.477	74.119	225.045	3	2.75	0.045874	1437.56

ZINC93275037	336.82	9.446	647.261	249.735	111.715	214.16	3	4.7	0.080776	601.66
ZINC93275037	336.82	8.295	644.509	245.597	117.681	209.579	3	4.7	0.062353	556.08
ZINC79074099	338.329	5.97	651.614	248.761	64.228	204.64	2	2.75	0.032929	1822.09
ZINC70763183	338.329	6.045	610.822	113.956	84.819	314.45	3	2	0.035098	992.18
ZINC70763183	338.329	5.884	596.897	117.457	82.117	299.807	3	2	0.033482	1160.25
ZINC72560748	338.368	3.171	630.968	104.35	129.33	397.288	3	5.75	0.009196	415.50
ZINC72560748	338.368	2.759	644.983	99.743	139.887	405.353	3	5.75	0.0069	297.88
ZINC32502283	337.377	12.438	608.766	197.11	104.799	306.857	2.25	4.75	0.144737	678.92
ZINC75137076	338.399	10.261	629.006	275.401	120.196	138.656	3	5.7	0.097146	504.02
ZINC75137076	338.399	10.693	649.552	257.937	143.017	153.886	3	5.7	0.104365	276.65
ZINC75137076	338.399	11.008	646.974	256.335	151.203	153.761	3	5.7	0.110926	232.63
ZINC75137076	338.399	10.253	629.258	275.441	119.884	139.224	3	5.7	0.096999	510.05
ZINC43360170	338.426	3.928	633.04	234.112	107.824	261.957	2	4	0.014032	636.17
ZINC43360170	338.426	9.732	643.575	233.816	103.864	261.607	2	4	0.085606	656.81
ZINC40097662	338.765	10.095	625.157	142.202	95.23	277.875	3	4.45	0.097628	857.90
ZINC40097662	338.765	11.693	624.787	141.276	95.28	278.491	3	4.45	0.131011	849.05
ZINC75414890	339.18	2.812	615.735	44.446	147.443	286.563	1.5	4	0.007786	245.02
ZINC91046473	339.753	7.9	581.683	227.318	109.822	136.999	2	5.2	0.062713	715.17
ZINC91046473	339.753	7.695	579.358	234.253	107.023	130.942	2	5.2	0.059322	696.81
ZINC91046473	339.753	7.707	578.994	234.835	106.831	130.022	2	5.2	0.059499	705.27
ZINC91046473	339.753	6.835	583.992	227.224	109.249	137.676	2	5.2	0.046912	729.46
ZINC44246717	340.359	13.24	649.88	143.314	159.135	300.41	2	5	0.158666	223.65
ZINC66952522	340.421	5.497	669.024	320.401	92.902	255.721	3	3.5	0.026253	875.71
ZINC66952522	340.421	8.218	631.619	332.988	71.999	226.632	3	3.5	0.059893	1425.85
ZINC77702068	341.796	3.104	623.674	54.675	119.736	377.616	3	4.7	0.009053	509.78
ZINC77702068	341.796	5.472	615.602	63.791	106.155	374.004	3	4.7	0.028237	701.76

Table S5. The ADME study carried out for database

Molecule	mol_MW	dipole	SASA	FOSA	FISA	PISA	dip^2/V	QPlogPw	QPlogPo/w
4a	279.33	8.68	534.30	21.33	154.66	229.93	0.09	14.50	1.33
4b	295.33	7.56	546.69	21.33	209.28	187.70	0.07	16.60	0.60
4c	309.36	7.39	571.89	114.51	154.61	174.39	0.06	14.73	1.45
4d	309.36	9.91	571.78	114.39	154.66	174.34	0.11	14.73	1.45
4e	325.36	8.61	584.39	114.47	205.92	135.62	0.08	16.83	0.75
4f	313.78	7.85	552.58	13.27	154.65	194.52	0.07	14.33	1.74
4g	313.78	7.34	558.26	21.33	154.66	182.38	0.06	14.26	1.81
4h	358.23	9.09	563.34	21.30	154.65	181.68	0.09	14.27	1.88
4i	358.23	7.46	563.30	21.34	154.66	181.65	0.06	14.27	1.88
4j	297.33	7.30	543.20	21.32	154.66	192.01	0.06	14.28	1.56
4k	324.33	7.09	571.82	12.43	246.24	184.77	0.05	15.67	0.71
4l	324.33	6.57	573.02	21.98	251.24	171.42	0.05	15.62	0.66
4m	322.40	8.93	610.12	156.37	154.64	170.73	0.08	15.05	1.81
4n	293.36	9.24	551.20	59.01	153.78	210.03	0.09	13.91	1.75
4o	307.39	9.63	582.56	146.70	153.78	153.71	0.10	13.60	2.04
4p	309.36	10.48	563.84	59.01	208.57	167.88	0.12	16.01	1.01
4q	323.39	10.50	585.36	162.54	143.92	150.52	0.11	14.04	1.91
4r	327.81	7.95	575.09	59.00	153.78	162.51	0.07	13.67	2.23
4s	372.26	8.07	580.13	59.00	153.78	161.78	0.07	13.68	2.31
4t	338.36	7.20	587.99	59.01	250.18	150.42	0.05	15.01	1.08
4u	355.43	9.93	623.03	0.00	135.54	359.86	0.09	14.76	2.99
4v	280.32	6.16	527.84	21.54	182.92	195.00	0.04	15.92	0.68
4w	269.30	8.40	496.90	29.44	156.53	182.55	0.09	14.62	0.73
4x	259.34	9.12	530.50	213.12	179.82	9.19	0.10	13.28	0.82
5a	320.34	10.65	563.77	0.00	241.69	193.71	0.12	16.15	0.68
5b	399.24	10.20	592.80	0.00	241.68	145.45	0.11	15.91	1.23
5c	365.34	10.98	604.12	0.00	339.08	136.66	0.12	17.28	0.02
